# *Neomonodictysaquatica* sp. nov. (Pleurotheciaceae) from a plateau lake in Yunnan Province, China

**DOI:** 10.3897/BDJ.10.e76842

**Published:** 2022-02-16

**Authors:** Si-Ping Huang, Dan-Feng Bao, Hong-Wei Shen, Hong-Yan Su, Zong-Long Luo

**Affiliations:** 1 College of Agriculture and Biological Sciences, Dali University, Dali, China College of Agriculture and Biological Sciences, Dali University Dali China; 2 Center of Excellence in Fungal Research, Mae Fah Luang University, Chiang Rai, Thailand Center of Excellence in Fungal Research, Mae Fah Luang University Chiang Rai Thailand; 3 Department of Entomology and Plant Pathology, Faculty of Agriculture, Chiang Mai University, Chiang Mai, Thailand Department of Entomology and Plant Pathology, Faculty of Agriculture, Chiang Mai University Chiang Mai Thailand; 4 School of Science, Mae Fah Luang University, Chiang Rai, Thailand School of Science, Mae Fah Luang University Chiang Rai Thailand

**Keywords:** new species, asexual morph, freshwater fungi, phylogeny, taxonomy

## Abstract

**Background:**

In this study, a new species *Neomonodictysaquatica* was collected from submerged decaying wood in Erhai Lake, Yunnan Province, China.

**New information:**

*Neomonodictysaquatica* is characterised by acrogenous, solitary, oval, dictyospores (most are transverse septum, occasionally vertical septum, in immaturity the septum is clear, but when mature, the conidia becomes darker so the septum is not clear), smooth-walled conidia. The immature conidia are usually hyaline to olivaceous and mature conidia are usually darkened to black, sometimes with one pale basal cell. Phylogenetic analyses of combined ITS and LSU sequence data showed that the new collection is distinct from other *Neomonodictys* species. Description and illustration are provided as well.

## Introduction

Pleurotheciales was introduced by Réblová et al. (2016), based on morphological characters and phylogenetic analyses. Members of Pleurotheciales are mostly saprobic on wood (Hyde et al. 2020) and some species have been identified as opportunistic human pathogens (Guarro et al. 2000, Chew et al. 2010, Réblová et al. 2020). Species of the order were collected on submerged decaying wood in lentic and lotic habitats in temperate, subtropical and tropical zones in Asia, Europe, Melanesia and North America (Matsushima 1971, Réblová et al. 2012, Réblová et al. 2016, Réblová et al. 2020, Hernández-Restrepo et al. 2017, Hyde et al. 2018, Hyde et al. 2020, Luo et al. 2018a).

Pleurotheciaceae is a single family of Pleurotheciales. It is typified by *Pleurothecium* with *P.recurvatum* as the type species (Morgan) Höhn (Réblová et al. 2016). Recently, Hyde et al. (2020) updated the phylogenetic tree for Pleurotheciales and introduced a new genus *Neomonodictys* Y.Z. Lu, C.G. Lin & K.D. Hyde. Currently, ten genera are accepted in this family (Réblová et al. 2016, Maharachchikumbura et al. 2016, Hernández-Restrepo et al. 2017, Hyde et al. 2020, Goh and Kuo 2021).

The monophyletic asexual genus *Neomonodictys* is established for a fungus (*Neomonodictysmuriformis*) collected from a freshwater habitat in Thailand, which is morphologically similar to members of *Monodictys* S. Hughes (Hyde et al. 2020). *Neomonodictys* is characterised by holoblastic, monoblastic, integrated, terminal, determinate conidiogenous cells and muriform, subglobose to globose, smooth-walled, pale brown to darkened to black conidia (Hyde et al. 2020).

In this study, the fungus was isolated from submerged decaying wood in Erhai Lake, Yunnan Province in China. The morphology and phylogeny show that our collection is distinct from related species. We provide detailed descriptions, illustrations for *Neomonodictysaquatica* and a synopsis table for the morphology comparison.

## Materials and methods

**Isolation and morphological examination**

Submerged decaying wood was collected from Erhai Lake, Dali City, Yunnan, China. The coordinates of sampling sites are 25°44′29.65″N, 100°09′49.33″E and at an altitude of 1966 m. Samples were returned to the laboratory in plastic bags. The samples were incubated in aseptic plastic boxes, lined with moistened tissue paper at room temperature for one week. Specimen observations and morphological studies were conducted following the protocols provided by Luo et al. (2018b).

Morphological observations were made by using a SMZ760 series stereomicroscope and photographed using a Nikon-80i microscope. The fungal structures were measured with Tarosoft (R) Image Frame Work programme and images were processed using Adobe Photoshop CS6 extended version 13.0 (Adobe Systems, USA). Single spore isolation was carried out following the method described in Chomnunti et al. (2014). Germinating conidia were transferred aseptically to PDA plates with 0.5 mg/l of Amoxicillin and incubated at room temperature under dark conditions. The colonies were checked every three days. A herbarium was deposited in the herbarium of Cryptogams Kunming Institute of Botany Academia Sinica (KUN-HKAS), Yunnan, China. Living cultures were deposited in Kunming Institute of Botany Culture Collection (KUNCC) and China General Microbiological Culture Collection Center (CGMCC). Facesoffungi numbers were registered as described in Jayasiri et al. (2015) and Index Fungorum numbers as in Index Fungorum(2021).

**Molecular Phylogenetic Analyses**

**DNA Sequencing and Sequence Alignment**

The appropriate fungal mycelium was scraped from the surface of colonies on Potato Dextrose Agar (PDA) plates with a scalpel into a 1.5 ml EP tube (Bao et al. 2018). Genomic DNA was extracted using the Trelief^TM^ Plant Genomic DNA Kit (Beijing TsingKe Biological Technology and Services Co. Ltd, China) according to the manufacturer’s protocols.

The primers ITS4/ITS5 for Internal transcribed spacer (ITS) and LR0R/LR5 for Large subunit ribosomal ribonucleic acid (LSU rRNA) were selected for PCR amplification (Vilgalys and Hester 1990). Polymerase Chain Reaction (PCR) mixture was performed in a 25 μl system reaction containing 9.5 μl ddH_2_O, 12.5 μl of 2 × Power Taq PCR Master Mix, 1 μl of DNA template and 1 μl of each primer (10 μM) (Wang et al. 2019). The PCR thermal cycles for amplification of the ITS gene region were as per Su et al. (2015) and the LSU gene followed Sun et al. (2020). PCR amplifications were confirmed on 1% agarose electrophoresis gels stained with ethidium bromide.

Sequences were assembled with BioEdit. Sequences with high similarity indices were determined from a BLAST search to find the closest matches with taxa in *Neomonodictys* and from recently published data (Ariyawansa et al. 2015, Wanasinghe et al. 2015, Hyde et al. 2019, Hyde et al. 2020). All consensus sequences and the reference sequences were aligned in MAFFT v. 7 (http://mafft.cbrc.jp/alignment/server/index.html, Katoh and Standley 2013). Aligned sequences of each gene region (ITS and LSU) were combined and manually improved using BioEdit v. 7.0.5.2 (Hall 1999). Ambiguous regions were excluded from the analyses and gaps were treated as missing data.

**Phylogenetic Analyses**

Maximum Likelihood analysis was performed in the CIPRES Science Gateway v.3.3 (Miller et al. 2010) using RAxML v. 8.2.8 as part of the “RAxML-HPC2 on XSEDE” tool (Stamatakis 2006, Stamatakis et al. 2008). The final ML search was conducted using the GTRGAMMA + I model estimated using MrModeltest 2.2 (Nylander 2004), with ML bootstrap support being calculated from 1000 bootstrap replicates.

Bayesian analysis was performed using MrBayes v. 3.1.2. (Ronquist and Huelsenbeck 2003). The model of each gene was estimated using MrModeltest 2.2 (Nylander 2004), with GTR + I + G model being the best-fit model of ITS and LSU for Bayesian analysis. Posterior Probabilities (PP) (Rannala and Yang 1996) were performed by Markov Chain Monte Carlo sampling (MCMC) in MrBayes v.3.1.2 (Liu et al. 2012). Six simultaneous Markov chains were run for 50 million generations and trees were sampled every 5000^th^ generation (resulting in 10,000 trees). The first 2000 trees, representing the burn-in phase of the analyses, were discarded and the remaining 8000 (post burning) trees were used for calculating posterior probabilities (PP) in the majority rule consensus tree (Cai et al. 2006, Liu et al. 2012).

Phylogenetic trees were visualised by FigTree v. 1.4.4 (Rambaut 2014) and edited in Microsoft Office PowerPoint 2016 (MicrosoftInc. United States). Newly-produced sequences in this study were submitted to GenBank (Table 1).

## Taxon treatments

### Neomonodictys aquatica

D.F. Bao, S.P. Huang & Z.L. Luo sp. nov.

A78A5B68-C181-5A92-892A-865AFBC15D3E

558842

Facesofungi number: FOF 10537

#### Materials

- **Type status:** Holotype. **Occurrence:** recordedBy: Longli Li; Siping Huang; **Taxon:** scientificName: *Neomonodictysaquatica*; kingdom: Fungi; phylum: Ascomycota; class: Sordariomycetes; order: Pleurotheciales; family: Pleurotheciaceae; genus: Neomonodictys; **Location:** waterBody: Erhai Lake; locality: Baitaiyi; verbatimElevation: 1966 m; locationRemarks: China, Yunnan Province, Dali, saprobic on submerged decaying wood in Erhai Lake; verbatimLatitude: 25 44 29.65N; verbatimLongitude: 100d 09' 49.33'' E; **Event:** year: 2020; habitat: freshwater, submerged decaying wood; **Record Level:** collectionID: 2EH 3-17-1 H; collectionCode: L127.

#### Description

**Figure 1.**
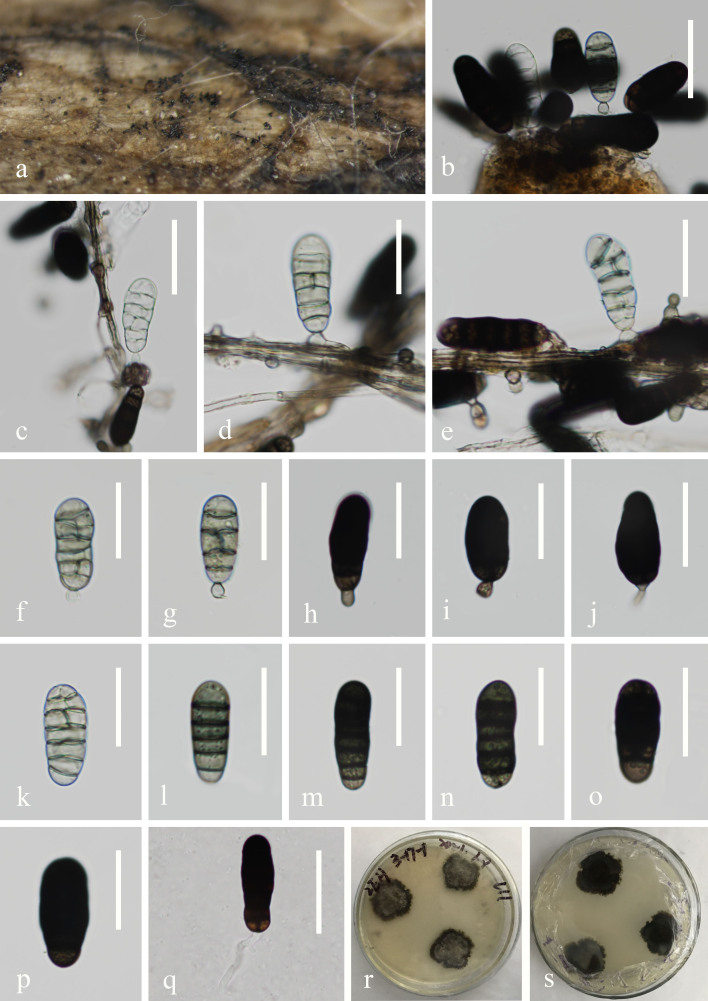
***Neomonodictysaquatica*** (KUN-HKAS 115806, holotype). **a** Colonies on submerged wood; **b-e** Conidiophores with conidia; **f-j** Conidiogenous cells with conidia; **k-p** Conidia; **q** Germinating conidium; **r, s** Colony on PDA. Scale bars: **b-c, e, k** = 25 μm; **d, f-i, l-p** = 20 μm; **j, q** = 30 μm.

**Sexual morph** Undetermined. **Asexual morph** Hyphomycetous (Fig. 1) sporodochia. Colonies on natural substratum superficial, scattered, black, glistening. Mycelium immersed in the substrate, composed of septate, smooth, thin-walled, light to dark brown, 2–3 μm wide hyphae. Conidiophores lacking. Conidiogenous cells short or occasionally missing, suborbicular, holoblastic, monoblastic, integrated, terminal, determinate, hyaline to pale brown. 3.7–6.4 × 2.9–4.7 μm (x̄ = 5.1 × 3.8 μm, n = 10). Conidia 23.1–29.5 × 8.5–11.5 μm (x̄ = 26 × 10 μm, n = 30), acrogenous, acrospore, oval, ellipsoidal to obovoid, muriform, smooth-walled, hyaline when young, becoming dark brown at maturity sometimes with one pale basal cell.

**Culture characteristics**: Conidia germinate on PDA in 36 h. Colonies growing on PDA, subglobose, with flat surface, edge jagged, reaching 3 cm long and 2.5 cm wide in 12 weeks at 28°C, dark grey in PDA medium. Mycelium superficial and partially immersed, branched, septate, hyaline to pale brown, smooth.

**Material examined**: China, Yunnan Province, Dali, sprobic on submerged decaying wood in Erhai Lake, September 2020, S. P. Huang, L-127 (KUN-HKAS 115806, holotype), ex-type living culture, KUNCC 21-10708 = CGMCC3.20681.

#### Etymology

Name reflects the aquatic habitat of this fungus

#### Notes

**Figure 2.**
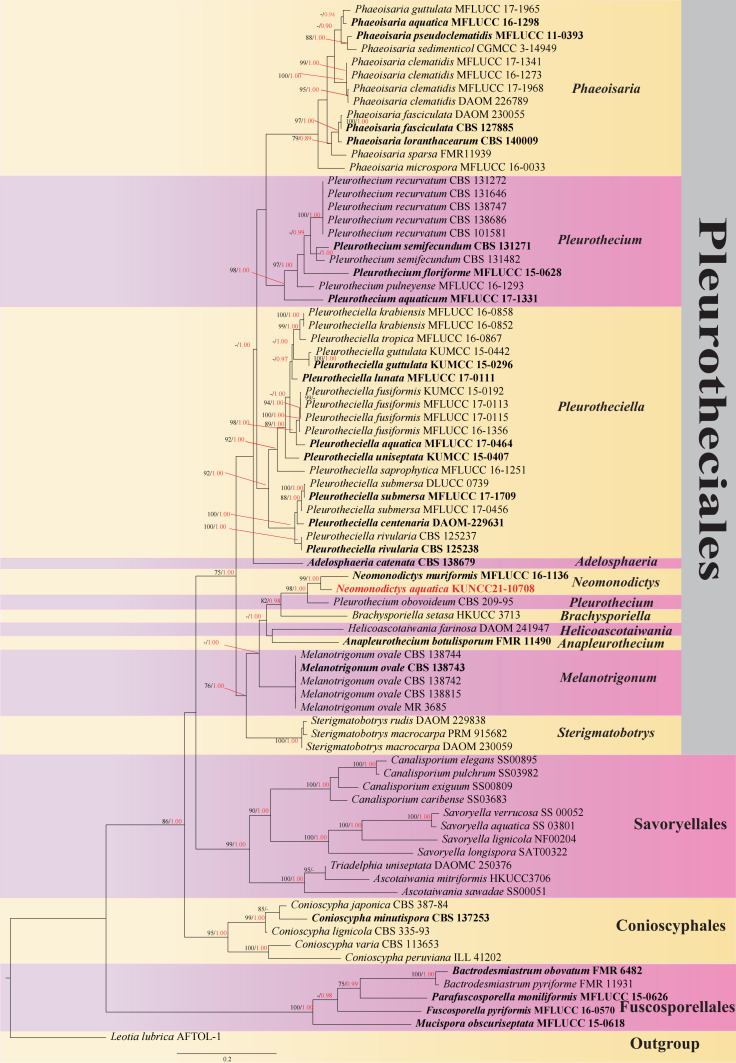
Phylogenetic tree based on RAxML, generated from a combined ITS and LSU dataset. Bootstrap support values for Maximum Likelihood (ML, black) higher than 75% and Bayesian posterior probabilities (BYPP, red) greater than 0.95 are indicated above the nodes as ML/PP. The tree is rooted to *Leotialubrica*. The type-derived sequences are indicated in bold and new isolates are in red. Bootstrap values for Maximum Likelihood (ML) equal to or greater than 75% and clade credibility values greater than 0.90 from Bayesian-inference analysis labelled on the nodes. Ex-type strains are in bold and black, the new isolate is indicated in bold and red. (Fig. 2).

Morphologically, *Neomonodictysaquatica* is easily distinguished from *N.muriformis*. *Neomonodictysmuriformis* has wider conidia than *N.aquatica* (15–25 vs. 8–12.2 μm). In addition, conidia of *N.aquatica* are oval or ellipsoidal to obovoid, while *N.muriformis* has subglobose to globose conidia. In the phylogenetic analysis, *N.aquatica* clustered with *N.muriformis* with strong support (99% ML and 1.00 PP) (Fig. 2). ITS comparison between our strain and MFLUCC 16-1136 revealed 57 bp difference in a total of 539 bp. LSU comparison between our strain and MFLUCC 16-1136 revealed 13 bp difference in total of 829 bp (Jeewon and Hyde 2016). Therefore, we introduce our new isolate as a new species.

## Analysis

**Phylogenetic analyses**

The phylogram generated from Maximum Likelihood analysis, based on combined ITS and LSU sequence data, represents Pleurotheciales and the closely related orders. Seventy-nine strains are included in the combined analyses, which comprise 2039 characters (ITS: 849 bp, LSU: 1190 bp) after aligning. *Leotiatubrica* (AFTOL-1) is the outgroup taxon in this phylogentic tree. The best RAxML tree with a final likelihood value of -12803.740107 is presented. The matrix had 698 distinct alignment patterns with 34.21% undetermined characters or gaps. Estimated base frequencies were as follows: A = 0.222096, C =0.295691, G = 0.272214, T = 0.209999; substitution rates AC = 1.588217, AG = 2.820721, AT = 2.535737, CG = 1.003016, CT = 5.905028, GT = 1.000000; gamma distribution shape parameter α = 0.570011.

In the phylogenetic analysis, our new isolate *Neomonodictysaquatica* clustered as a sister taxon with *N.muriformis* with strong bootstrap support (99 ML/1.00 PP, Fig. 2).

## Discussion

Up to now, two species are accepted in *Neomonodictys*, including the newly-introduced species. Both of them are collected from submerged wood in freshwater habitats (Hyde et al. 2020) and only asexual morphs are reported. Morphologically, *Neomonodictys* is similar to *Monodictys* in having solitary, dictyospores conidia and monoblastic, hyaline to brown conidiogenous cells (Ellis 1971, Seifert et al. 2011). Compared with the diaphragms of them, *Neomonodictysaquatica* have a mostly transverse septum, less of the vertical septum, but the transerve and vertical septa of *N.muriformis* are evenly distributed. The significant difference between *Neomonodictys* and *Monodictys* is conidiophores, which are shorter than in the former (Kukwa and Diederich 2005). Phylogenetically, they are distinct (Hyde et al. 2020). In the phylogenetic analysis, *Monodictys* was placed in Dothideomycetes (Day et al. 2006, Seifert et al. 2011, Wijayawardene et al. 2020), while *Neomonodictys* was placed in Sordariomycetes (Hyde et al. 2020).

## Supplementary Material

XML Treatment for Neomonodictys aquatica

## Figures and Tables

**Table 1.** Isolates and sequences used in this study (newly-generated sequences are indicated in bold and with “*” after species name, the type strains are in bold).

**Taxon**	**Strain**	**GenBank Accession No.**	
		**ITS**	**LSU**
***Adelosphaeriacatenata***	**CBS 138679**	**NR**_**145396**	**MH877664**
***Anapleurotheciumbotulisporum***	**FMR 11490**	**NR_153582**	KY853483
*Ascotaiwaniamitriformis*	HKUCC3706	−	AF132324
*Ascotaiwaniasawadae*	SS00051	HQ446340	HQ446363
***Bactrodesmiastrumobovatum***	**FMR 6482**	**NR_152537**	FR870266
*Bactrodesmiastrumpyriforme*	FMR 11931	HE646636	HE646637
*Brachysporiellasetasa*	HKUCC 3713	−	AF132334
*Canalisporiumcaribense*	SS03683	GQ390284	GQ390269
*Canalisporiumelegans*	SS00895	GQ390286	GQ390271
*Canalisporiumexiguum*	SS00809	GQ390296	GQ390281
*Canalisporiumpulchrum*	SS03982	GQ390292	GQ390277
*Conioscyphajaponica*	CBS 387.84	−	AY484514
*Conioscyphalignicola*	CBS 335.93	−	AY484513
***Conioscyphaminutispora***	**CBS 137253**	**NR_137847**	**NG_066275**
*Conioscyphaperuviana*	ILL 41202	−	**NG_058867**
*Conioscyphavaria*	CBS 113653	−	AY484512
***Fuscosporellapyriformis***	**MFLUCC 16-0570**	**NR_152555**	**NG_059711**
*Helicoascotaiwaniafarinosa*	DAOM 241947	JQ429145	JQ429230
*Melanotrigonumovale*	MR 3685	KT278726	KT278712
*Melanotrigonumovale*	CBS 138744	KT278725	KT278710
*Melanotrigonumovale*	CBS 138815	KT278722	KT278711
***Melanotrigonumovale***	**CBS 138743**	**NR_145397**	**NG_058197**
*Melanotrigonumovale*	CBS 138742	KT278723	KT278708
***Mucisporaobscuriseptata***	**MFLUCC 15-0618**	**NR_152556**	**NG_059709**
***Neomondictysaquatica*** *	**KUNCC21-10708**	**MZ686200**	**OK245417**
***Neomonodictysmuriformis***	**MFLUCC 16-1136**	**NR_168231**	**NG_068916**
***Parafuscosporellamoniliformis***	**MFLUCC 15-0626**	**NR_152557**	**NG_059710**
***Phaeoisariaaquatica***	**MFLUCC 16-1298**	**NR_160592**	**NG_066194**
*Phaeoisariaclematidis*	MFLUCC 16-1273	MF399229	MF399246
*Phaeoisariaclematidis*	DAOM 226789	JQ429155	JQ429231
*Phaeoisariaclematidis*	MFLUCC 17-1968	MG837022	MG837017
*Phaeoisariaclematidis*	MFLUCC 17-1341	MF399230	MF399247
*Phaeoisariafasciculata*	DAOM 230055	KT278720	KT278706
***Phaeoisariafasciculata***	**CBS 127885**	**NR_145395**	**NG_064241**
*Phaeoisariaguttulata*	MFLUCC 17-1965	MG837021	MG837016
***Phaeoisarialoranthacearum***	**CBS 140009**	**NR_56593**	**NG_064294**
***Phaeoisariapseudoclematidis***	**MFLUCC 11-0393**	**NR_155648**	**NG_059559**
*Phaeoisariasedimenticol*	CGMCC 3.14949	MK878380	MK835851
*Phaeoisariasparsa*	FMR11939	HF677179	HF677185
*Phaeoisariamicrospora*	MFLUCC 16-0033	MF671987	−
***Pleurotheciellaaquatica***	**MFLUCC 17-0464**	**NR_160591**	**NG_066193**
***Pleurotheciellacentenaria***	**DAOM 229631**	**NR_111709**	**NG_060098**
***Pleurotheciellalunata***	**MFLUCC 17-0111**	**NR_160593**	**NG_066195**
***Pleurotheciellarivularia***	**CBS 125238**	**NR_111711**	**NG_057950**
*Pleurotheciellarivularia*	CBS 125237	JQ429161	JQ429233
*Pleurotheciellafusiformis*	KUMCC 15-0192	MF399234	MF399251
*Pleurotheciellafusiformis*	MFLUCC 17-0113	MF399233	MF399250
*Pleurotheciellafusiformis*	MFLUCC 17-0115	MF399232	MF399249
*Pleurotheciellafusiformis*	MFLUCC 16-1356	MF399235	MF399252
*Pleurotheciellaguttulata*	KUMCC 15-0442	MF399239	MF399256
***Pleurotheciellaguttulata***	**KUMCC 15-0296**	**NR_160594**	**NG_066399**
*Pleurotheciellakrabiensis*	MFLUCC 16-0852	MG837018	MG837013
*Pleurotheciellakrabiensis*	MFLUCC 16-0858	MG837019	MG837014
***Pleurotheciellasaprophytica***	**MFLUCC 16-1251**	**NR_160595**	**NG_066196**
***Pleurotheciellasubmersa***	**MFLUCC 17-1709**	**NR_160596**	MF399260
*Pleurotheciellasubmersa*	DLUCC 0739	MF399242	MF399259
*Pleurotheciellasubmersa*	MFLUCC 17-0456	MF399244	MF399261
*Pleurotheciellatropica*	MFLUCC 16-0867	MG837020	MG837015
*Pleurotheciellauniseptata*	KUMCC 15-0407	MF399231	MF399248
***Pleurotheciumaquaticum***	**MFLUCC 17-1331**	**NR_160597**	**NG_066197**
***Pleurotheciumfloriforme***	**MFLUCC 15-0628**	**NR_156614**	**NG_059791**
*Pleurotheciumobovoideum*	CBS 209.95	EU041784	EU041841
*Pleurotheciumpulneyense*	MFLUCC 16-1293	−	MF399262
*Pleurotheciumrecurvatum*	CBS 138686	KT278727	KT278715
*Pleurotheciumrecurvatum*	CBS 138747	KT278728	KT278714
*Pleurotheciumrecurvatum*	CBS 131646	JQ429150	JQ429236
*Pleurotheciumrecurvatum*	CBS 131272	JQ429149	JQ429237
*Pleurotheciumrecurvatum*	CBS 101581	JQ429148	−
*Pleurotheciumsemifecundum*	CBS 131482	JQ429158	JQ429239
***Pleurotheciumsemifecundum***	**CBS 131271**	**NR_111710**	**NG_057951**
*Savoryellaaquatica*	SS 03801	HQ446349	HQ446372
*Savoryellalignicola*	NF00204	HQ446357	HQ446378
*Savoryellalongispora*	SAT00322	HQ446359	HQ446380
*Savoryellapaucispora*	SAT00866	−	−
*Savoryellaverrucosa*	SS 00052	HQ446353	HQ446374
*Sterigmatobotrysmacrocarpa*	PRM 915682	JQ429153	_
*Sterigmatobotrysmacrocarpa*	DAOM 230059	−	GU017316
*Sterigmatobotrysrudis*	DAOM 229838	JQ429152	JQ429241
*Triadelphiauniseptata*	DAOMC 250376	−	KT278718
